# Cross-cultural adaptation and reproducibility of the EPIC-Norfolk food frequency questionnaire in young people living in Croatia

**DOI:** 10.3325/cmj.2024.65.20

**Published:** 2024-02

**Authors:** Erna Davidović Cvetko, Ines Drenjančević, Ivana Jukić, Ana Stupin, Petar Šušnjara, Martina Vulin, Martina Kos, Matea Lukić, Lorena Stanojević

**Affiliations:** 1 University of Applied Sciences Lavoslav Ružička in Vukovar, Vukovar, Croatia; 2 Department of Physiology and Immunology, Faculty of Medicine in Osijek, J.J. Strossmayer University of Osijek, Osijek, Croatia; 3 Department of Obstetrics and Gynecology. University Hospital Osijek, Osijek, Croatia; 4 Clinic of Pediatrics, University Hospital Osijek, Osijek, Croatia

## Abstract

**Aim:**

To translate and adapt the European Prospective Investigation of Cancer (EPIC)-Norfolk food frequency questionnaire (FFQ) for use in Croatia, and to assess the reliability and reproducibility of the Croatian version of the EPIC-Norfolk FFQ.

**Methods:**

Translation and cross-cultural adaptation were performed according to published recommendations. Reliability was assessed in 140 respondents (61 men; age range 8-40 years) divided into three groups: young adults, pregnant women, and children and adolescents. Reproducibility was assessed in the group of young adults (32/61 men), who completed the questionnaire on two occasions three months apart.

**Results:**

The EPIC-Norfolk FFQ showed good reliability (Cronbach alpha = 0.874). Most nutrient intakes showed good reproducibility (intraclass correlation coefficient [ICC] between 0.7 and 0.9). Poor reproducibility was observed for alcohol (ICC = 0.337), and moderate reproducibility was observed for beta-carotene (ICC = 0.692) and total carbohydrates (ICC = 0.698). Nutrient intakes measured by FFQ on two occasions did not significantly differ.

**Conclusion:**

The Croatian version of the EPIC-Norfolk FFQ can be a useful tool for assessing dietary intakes in young people in Croatia and possibly in neighboring countries with similar languages and dietary habits.

Nutritional habits are among the most important lifestyle characteristics related to health. Poor dietary habits are associated with a range of chronic diseases and can contribute to mortality from non-communicable diseases (1).

One of the greatest challenges in nutritional epidemiology is to accurately assess dietary intake (2). Food frequency questionnaires (FFQ) are nutritional assessment tools used to estimate the individual food intake by inquiring about the frequency and quantity of consumption of food items or food groups (3). They offer a low-cost, available, and simple way of recording eating habits, which makes them an instrument of choice in epidemiological studies investigating the links between dietary habits and health or disease risks (4). Besides a structured food list, FFQ usually consist of a food composition database, which compiles the foods commonly consumed in the study population and allows the calculation of nutrient intake (5). The food list should be appropriate for the study group, because dietary habits differ according to ethnicity, economic status, culture, and location (6). It should also precisely specify the portion size to avoid systematic errors in the assessment of nutritional intake (7). One of the easiest FFQs to use in terms of administration and data processing is the European Prospective Investigation of Cancer (EPIC)-Norfolk FFQ. This FFQ is freely available online and provides software for data processing (8). In Croatia, there are available questionnaires for data collection from special populations or assessment of the intake of specific nutrients. However, most of them are constructed to be used by nutritionists and dietitians, who are much more familiar with food composition than are epidemiologists assessing food-related risks. There is no questionnaire in the Croatian language that is easy to administer and that provides software for data processing. Although the EPIC-Norfolk FFQ was designed primarily for the adult population in the UK, the food list is similar to that from the national food consumption survey in Croatia conducted in 2011 and 2012 (9). The EPIC Norfolk FFQ was validated for children and adolescents in Italy (10) and in young adults in Lebanon (11).

The aim of this study was to translate and adapt the EPIC-Norfolk FFQ for use in Croatia, and to assess the reliability and reproducibility of the Croatian version of the FFQ.

## Respondents and methods

### Study design

The study consisted of two phases: 1) translation and cross-cultural adaptation of the EPIC-Norfolk FFQ and 2) administration of the Croatian version of the questionnaire on two occasions, three months apart, for the reproducibility analysis.

### The EPIC-Norfolk food frequency questionnaire

The EPIC-Norfolk FFQ was created in 1988. It was validated and used to assess dietary intake on a large sample in the EPIC study (12). This semiquantitative questionnaire is designed to record the average intake of foods during the previous year. It consists of two parts. Part 1 is a food list consisting of 130 items, which were classified in 10 groups as follows: 1) meat and fish, 2) bread and savory biscuits, 3) cereals, 4) potatoes, rice, and pasta, 5) dairy products and fats, 6) sweets and snacks, 7) soups, sauces, and spreads, 8) drinks, 9) fruits, and 10) vegetables. The portion size was specified for each item. Respondents select the frequency of consumption for each food item from nine frequency categories (from “never or less than once a month” to “six or more times a day”). Part 2 consists of additional questions aimed at obtaining more details about food items from Part 1, particularly breakfast cereals, the type and quantity of milk consumed, the type of fat used in cooking, the amount of visible fat on meat consumed, and supplements used during the previous year. The EPIC-Norfolk FFQ has been validated and widely used (13-15). A copy of the questionnaire and the instructions for completing and coding are available for download from the website of the EPIC study.

### Translation and cross-cultural adaptation

The questionnaire was translated and cross-culturally adapted according to WHO instructions (16) and a study by Guillemin et al (17). Two professional translators independently translated the questionnaire from English to Croatian. This process produced two Croatian-language versions of the questionnaire. The versions were compared, and since there was no considerable difference in translation, a unique version was produced by the agreement of the two translators. The questionnaire was then back-translated to English by a third translator, a native English speaker, who was not familiar with the original English version of the questionnaire. Back translation yielded an English version of FFQ, which was not semantically or stylistically different from the original EPIC-Norfolk FFQ.

A focus group of expert nutritionists and dietitians was formed to discuss every item in the questionnaire, the adequacy of the food list for the composition of usual dishes in Croatian cuisine, and the adequacy of portion sizes provided by the questionnaire for the usual nutritional use at different ages. They agreed that portion sizes were adequate for every stage of life because they were small enough for children in Croatia and could be adjusted by the frequency of consumption. For example, if a food item is consumed in a twice-larger size than the portion size provided in the FFQ, the frequency of use can be multiplied by two. Brand names in the English FFQ were replaced with Croatian brand-name products that had the same or very similar nutrient composition. The brand-name products were described in detail to facilitate the use of the questionnaire for those who are unfamiliar with particular brand names. We aimed to make the food list as similar in nutrient composition as possible to the English version to be able to use the data processing software for calculating the nutrient intake. A small focus group (N = 15) was asked to fill in the questionnaire and assess if it was understandable and adequately adapted, and if it included adequate food items in the food list. Their suggestions were accepted (mainly about clarifying the food list by including additional colloquial terms for some foods in the food list), and a final version of FFQ in Croatian was produced (The questionnaire in Croatian is available upon request).

### Administration of the Croatin FFQ for reliability and reproducibility analysis

The Croatian questionnaire was administered to 140 participants who volunteered for this study. The need for volunteers was advertised by departmental staff during communication with students and hospital staff with patients. Most of the participants lived in the eastern parts of Croatia: Slavonia, Baranja, and Srijem. To test the reliability of the questionnaire for different populations, we divided the sample into three groups: young adults of participants of both sexes (N = 61); pregnant women (N = 27); and children and adolescents of both sexes (N = 52). The questionnaire was self-administered and paper-based. Children were helped by their parents/caregivers to fill in the questionnaire. We believe that this did not influence answers, since it is usual for children in Croatia to have all their meals at home with parents present. For the purpose of reproducibility analysis, the questionnaire was re-administered three months later to 61 participants from the young adults group. Young adults were selected for reproducibility analysis as they have the most stable nutritional habits among the included groups. The study protocol complied with the latest revision of the Declaration of Helsinki and the national legislation. The study was approved by the Ethics Committee of the University of Osijek, Faculty of Medicine.

### Statistical analysis

Descriptive results were expressed as means and standard deviations, or as percentages and frequencies. To test the reliability of the questionnaire, Cronbach's alpha coefficient was used to assess internal consistency, with alpha equal to or greater than 0.70 considered satisfactory (18). To determine the reproducibility of the questionnaire, we checked for agreement between the first and second administration of FFQ (FFQ1 and FFQ2) by using Spearman correlation and intra-class correlation coefficients (ICC) of nutrient intake for all the assessed nutrients (19). The difference in mean nutrient intakes between FFQ1 and FFQ2 was assessed with a paired *t* test on log transformed data. Bland-Altman plots were used to illustrate the degree of agreement between the intakes of selected nutrients assessed by FFQ1 and FFQ2. The difference between FFQ1 and FFQ2 was plotted against the mean intake of two measurements (FFQ1 and FFQ2). Statistical analysis was performed with SPSS 20.0 (IBM Corp., Armonk, NY, USA). The level of statistical significance was set at *P* = 0.05.

## Results

The final version of the FFQ was very similar to the original EPIC-Norfolk FFQ. Since no difference in nutrient composition was found between the foods listed in the Croatian FFQ compared with the original English FFQ, the nutrient intake was calculated with the FETA software (8). The intakes of 60 nutrients and food types were calculated. The Cronbach alpha for the results of FFQ was 0.874 (95% CI 0.839-0.905).

A total of 140 respondents filled in the FFQ (Table 1). The mean intake of nutrients and food types for the whole sample and for the subgroups is presented in Table 2. The Cronbach alpha values for the subgroups were as follows: young adults - 0.848 (95% CI 0.789-0.898); pregnant women - 0.843 (95% CI 0.745-0.917), children and adolescents - 0.885 (95% CI 0.810-0.941).

**Table 1:** General characteristics of respondents included in reliability testing of the translated EPIC-Norfolk Food Frequency Questionnaire

	Sex, n (%)		Age (years)		
	male	female	Min	Max	Mean ± SD
**Young adults** **(N = 61)**	32 (52)	29 (48)	18	27	21 ± 2.13
**Pregnant women** **(N = 27)**	0	27 (100)	22	40	30.3 ± 4.07
**Children and adolescents** **(N = 52)**	29 (56)	23 (44)	8	17	14.67 ± 2.42
**Total (N = 140)**	61 (44)	79 (56)	8	40	21.8 ± 6.05

**Table 2:** Mean daily intakes of nutrients and food types for the groups of young adults, pregnant women, and children and adolescents

Nutrient or a food type	Unit	Young adults (N = 61)		Pregnant women (N = 27)		Children and adolescents (N = 52)		Total (N = 140)	
		mean	SD	mean	SD	mean	SD	mean	SD
**Energy**	**kcal**	2375.7	992.9	1.992.9	712.6	4948.1	1994.8	2.792.9	1696.9
**Meat and meat products**	**g**	205.0	145.8	187.3	171.4	443.5	209.6	247.7	187.8
**Fish & fish products**	**g**	39.0	33.6	29.0	24.9	62.5	31.4	44.5	42.1
**Milk and milk products**	**g**	481.2	239.8	390.1	241.7	370.2	270.8	426.2	244.9
**Soups & sauces**	**g**	189.1	168.0	144.5	85.1	192.7	57.4	169.3	120.9
**Sugars; preserves and snacks**	**g**	45.7	34.2	50.1	50.5	120.3	43.9	64.1	52.9
**Vegetables**	**g**	187.4	167.6	227.6	97.7	293.1	114.7	202.8	133.4
**Fruit**	**g**	327.9	396.1	423.9	344.6	191.8	167.3	279.6	308.1
**Alcoholic beverages**	**g**	63.2	117.4	13.6	38.9	2.9	8.5	29.3	71.1
**Non-alcoholic beverages**	**g**	446.7	335.3	424.9	196.3	500.1	242.0	447.6	267.9
**Cereals and cereal products**	**g**	267.2	168.1	237.9	127.5	669.9	289.0	337.1	255.5
**Eggs and egg dishes**	**g**	28.6	43.2	26.3	25.2	26.3	23.9	25.4	29.5
**Fats and oils**	**g**	20.3	19.6	14.6	9.1	73.6	38.3	32.2	34.4
**Nuts and seeds**	**g**	5.0	9.8	9.2	8.1	6.9	14.2	6.3	10.1
**Potatoes. rice and pasta**	**g**	90.6	77.5	65.4	43.2	289.5	169.8	127.5	129.3
**Fat - total**	**g**	96.3	44.8	81.8	33.7	247.3	102.7	125.4	90.7
**Monounsaturated fatty acids (total)**	**g**	34.9	17.1	29.6	13.5	91.7	38.6	46.1	34.2
**Polyunsaturated fatty acids (total)**	**g**	17.7	9.7	15.2	7.5	39.9	15.5	21.6	14.7
**Saturated fatty acids (total)**	**g**	35.1	16.2	29.8	10.9	94.1	40.2	46.7	34.8
**Cholesterol**	**mg**	451.7	237.9	356.2	129.9	863.1	374.5	509.6	315.6
**Carbohydrate - total**	**g**	279.5	124.8	238.7	94.5	535.3	215.2	316.8	183.0
**Carbohydrate - sugars (total)**	**g**	143.5	75.9	131.1	57.8	210.8	84.3	149.8	77.2
**Carbohydrate - fructose**	**g**	26.6	19.6	26.3	12.7	31.9	12.8	25.7	15.2
**Carbohydrate - galactose**	**g**	0.6	0.9	0.8	1.01	0.4	0.3	0.5	0.7
**Carbohydrate - glucose**	**g**	23.5	17.0	23.9	10.4	34.7	13.1	24.8	14.3
**Carbohydrate - lactose**	**g**	24.0	11.4	19.4	10.7	23.4	14.0	22.3	11.8
**Carbohydrate - maltose**	**g**	2.8	1.7	2.6	2.4	8.3	3.5	3.9	3.3
**Carbohydrate - starch**	**g**	131.8	70.7	103.8	51.2	319.9	137.0	162.5	118.3
**Carbohydrate - sucrose**	**g**	60.7	38.2	55.1	37.4	103.2	43.5	66.8	42.5
**Englyst fiber – non starch polysaccharides (NSP)**	**g**	17.1	10.4	17.7	6.5	27.7	11.6	18.4	10.3
**Protein**	**g**	105.6	47.2	88.9	25.6	178.1	70.9	115.5	59.2
**Alpha carotene**	**µg**	403.3	343.2	660.4	363.2	572.1	390.5	469.0	360.4
**Beta carotene**	**µg**	2525.6	1686.5	3414.3	1388.0	3.904.2	1865.9	2.876.8	1659.1
**Carotene - total (carotene equivalents)**	**µg**	3016.7	1927.8	3971.8	1588.2	4.552.8	2272.0	3.378.5	1933.7
**Vitamin A - retinol**	**µg**	1542.1	2007.2	1032.9	1082.4	4769.8	4496.4	2154.5	3065.9
**Vitamin A - retinol equivalents**	**µg**	2048.2	2060.4	1699.6	1226.4	5548.2	4833.6	2724.9	3235.1
**Vitamin B2 - riboflavin**	**mg**	2.4	0.9	1.9	0.7	3.6	1.7	2.5	1.3
**Vitamin B1 - thiamin**	**mg**	1.8	0.8	1.7	0.6	3.2	1.2	2.0	1.0
**Vitamin B12 - cobalamin**	**µg**	9.6	6.4	7.3	4.5	22.2	16.3	11.9	11
**Vitamin B6 - pyridoxine**	**mg**	2.4	1.1	2.2	0.7	4.2	1.7	2.7	1.4
**Total folate**	**µg**	303.2	159.7	274.1	87.3	425.9	170.1	307.1	148.7
**Niacin**	**mg**	28.0	13.1	24.6	6.8	43.7	16.7	29.9	14.6
**Vitamin C - ascorbic acid**	**mg**	137.9	124.2	162.1	89.7	173.7	72.9	138.5	94.2
**Vitamin D - ergocalciferol**	**µg**	3.4	1.9	2.7	1.2	7.2	3.2	4.1	2.8
**Vitamin E - alpha tocopherol equivalents**	**mg**	14.7	8.4	12.9	6.7	29.5	11.5	17.4	11.1
**Zinc**	**mg**	11.4	4.8	9.6	3.0	21.5	9.4	12.9	7.3
**Nitrogen**	**g**	17.0	7.6	14.4	4.1	28.9	11.6	18.7	9.7
**Selenium**	**µg**	79.9	34.1	69.8	25.6	138.7	56.2	89.1	47.3
**Phosphorus**	**mg**	1702.1	659.5	1455.8	415.5	2740.4	1053.9	1833.4	859.6
**Magnesium**	**mg**	322.3	149.7	311.8	88.5	507.4	196.5	347.8	163.9
**Manganese**	**mg**	2.9	1.8	2.9	1.2	4.5	1.8	3.1	1.7
**Sodium**	**mg**	3698	1616.5	3084.9	1208.7	7564.1	2991.7	4268.4	2580.9
**Iodine**	**µg**	184.3	72.0	152.6	59.6	256.3	106.7	189.0	89.2
**Potassium**	**mg**	3893.9	1810.8	3603.9	1031.8	6531.3	2696.1	4241.8	2160.1
**Calcium**	**mg**	1130.9	395.2	957.2	342.1	1531.8	595.5	1146.3	478.7
**Chloride**	**mg**	5451.8	2414.8	4634.6	1792.8	10936.2	4321.1	6260.7	3715.8
**Copper**	**mg**	1.8	1.2	1.4	0.7	4.3	2.8	2.2	2
**Iron**	**mg**	11.9	5.3	10.4	3.6	23.6	10.4	13.7	8.2

**Figure 1:**
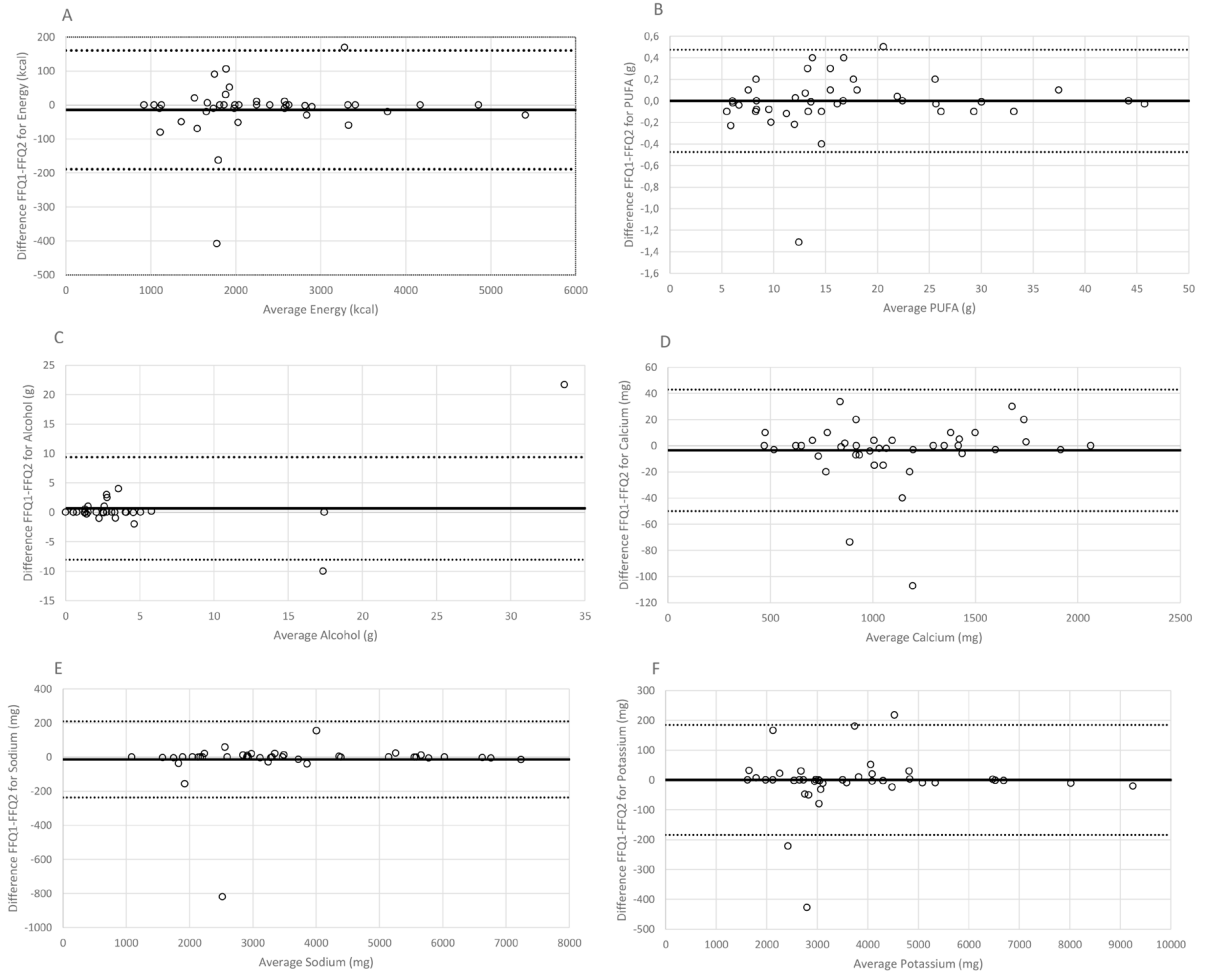
Bland-Altman plots show differences between two administrations of the Croatian version of the EPIC-Norfolk Food Frequency Questionnaire (FFQ1 and FFQ2) against their mean value ([FFQ1+FFQ2]/2) for daily intakes of (**A**) energy (kcal); (**B**) polyunsaturated fatty acids (PUFA) (g); (**C**) alcohol (g); (**D**) calcium (mg); (**E**) sodium (mg); and (**F**) potassium (mg). Solid lines represent the mean difference in nutrient intake between two administrations of the FFQ, while dashed lines represent 1.96 standard deviation (SD) of that difference.

The correlations between FFQ1 and FFQ2 in all nutrient intakes were significant. ICC values for most of the nutrient intakes were between 0.75 and 0.9. Moderate reproducibility was found for beta carotene (ICC = 0.684-0.695) and total carbohydrates (ICC = 0.666-0.690). ICC was low (0.337) only for alcohol intake (Table 3) (20). The Bland-Altman plots for selected nutrients: energy, calcium, potassium, sodium, alcohol, and polyunsaturated fatty acids showed a small mean difference between FFQ1 and FFQ2 (Figure 1). The mean differences in nutrient intake between FFQ1 and FFQ2 were not significant (Table 4).

**Table 3:** Results of reproducibility analysis: Spearman correlation coefficients and intraclass correlation coefficients (ICC) in two administrations of the food frequency questionnaire in 61 young adults. All the correlations were statistically significant

Nutrient	Spearman rho	ICC	95% confidence interval for ICC	
			lower bound	upper bound
**Energy**	0.840	0.861	0.829	0.879
**Fat (total)**	0.935	0.865	0.836	0.881
**Monounsaturated fatty acids (total)**	0.934	0.864	0.834	0.880
**Polyunsaturated fatty acids (total)**	0.928	0.856	0.819	0.876
**Saturated fatty acids (total)**	0.916	0.872	0.849	0.885
**Cholesterol**	0.981	0.874	0.852	0.886
**Carbohydrate (total)**	0.952	0.682	0.666	0.690
**Carbohydrate - sugars (total)**	0.948	0.884	0.871	0.891
**Carbohydrate - fructose**	0.918	0.848	0.805	0.872
**Carbohydrate - galactose**	0.895	0.855	0.818	0.876
**Carbohydrate - glucose**	0.907	0.852	0.812	0.874
**Carbohydrate - lactose**	0.935	0.808	0.735	0.849
**Carbohydrate - maltose**	0.913	0.843	0.795	0.868
**Carbohydrate - starch**	0.965	0.888	0.878	0.893
**Carbohydrate - sucrose**	0.958	0.886	0.874	0.892
**Englyst fiber – non starch polysaccharides**	0.952	0.886	0.875	0.893
**Alcohol**	0.922	0.337	0.036	0.581
**Protein**	0.944	0.868	0.841	0.882
**Alpha carotene**	0.959	0.785	0.771	0.791
**Beta carotene**	0.970	0.692	0.684	0.695
**Carotene - total (carotene equivalents)**	0.971	0.791	0.783	0.795
**Vitamin A - retinol**	0.918	0.790	0.704	0.839
**Vitamin B2 - riboflavin**	0.873	0.817	0.751	0.854
**Vitamin B1 - thiamin**	0.913	0.871	0.847	0.884
**Vitamin B12 - cobalamin**	0.846	0.766	0.664	0.826
**Vitamin B6 - pyridoxine**	0.931	0.867	0.839	0.882
**Total folate**	0.949	0.887	0.875	0.893
**Niacin**	0.923	0.859	0.825	0.878
**Vitamin C - ascorbic acid**	0.922	0.885	0.872	0.892
**Vitamin D - ergocalciferol**	0.936	0.858	0.823	0.877
**Vitamin E - alpha tocopherol equivalents**	0.942	0.875	0.855	0.886
**Zinc**	0.927	0.854	0.815	0.875
**Nitrogen**	0.947	0.868	0.842	0.883
**Selenium**	0.941	0.863	0.833	0.880
**Phosphorus**	0.957	0.881	0.866	0.890
**Magnesium**	0.956	0.882	0.866	0.890
**Manganese**	0.941	0.887	0.876	0.893
**Sodium**	0.932	0.850	0.809	0.873
**Iodine**	0.978	0.884	0.871	0.891
**Potassium**	0.746	0.875	0.854	0.886
**Calcium**	0.978	0.784	0.769	0.791
**Chloride**	0.921	0.853	0.814	0.874
**Copper**	0.854	0.810	0.738	0.851
**Iron**	0.931	0.931	0.873	0.962

**Table 4:** Results of the reproducibility analysis: mean daily nutrient intakes estimated by two administrations of the food frequency questionnaire (FFQ1 and FFQ2) in a group of young adults (N = 61)

Nutrient	Unit	FFQ1		FFQ2		P*
		mean	SD	mean	SD	
**Energy**	**kcal**	2375.7	992.9	2389.9	979.9	0.955
**Fat - total**	**g**	96.3	44.8	96.4	44.8	0.939
**Monounsaturated fatty acids (total)**	**g**	34.9	17.1	34.8	17.0	0.897
**Polyunsaturated fatty acids (total)**	**g**	17.7	9.7	17.9	9.7	0.674
**Saturated fatty acids (total)**	**g**	35.1	16.2	35.1	16.3	0.984
**Cholesterol**	**mg**	451.7	237.9	454.2	239.1	0.744
**Carbohydrate - total**	**g**	279.5	124.8	281.1	123.1	0.631
**Carbohydrate - sugars (total)**	**g**	143.5	75.9	143.6	75.0	0.964
**Carbohydrate - fructose**	**g**	26.6	19.6	26.3	19.2	0.742
**Carbohydrate - galactose**	**g**	0.6	0.9	0.7	0.9	0.063
**Carbohydrate - glucose**	**g**	23.5	17.0	23.1	15.4	0.575
**Carbohydrate - lactose**	**g**	24.0	11.4	24.5	11.9	0.467
**Carbohydrate - maltose**	**g**	2.8	1.7	2.8	1.8	0.717
**Carbohydrate - starch**	**g**	131.8	70.7	133.1	70.4	0.387
**Carbohydrate - sucrose**	**g**	60.7	38.2	60.5	38.1	0.804
**Englyst Fiber – non-starch polysaccharides**	**g**	17.1	10.4	17.1	10.3	0.878
**Protein**	**g**	105.6	47.2	106.1	45.4	0.755
**Alcohol**	**g**	4.4	8.3	3.7	5.9	0.116
**Alpha carotene**	**µg**	403.3	343.2	393.2	339.6	0.108
**Beta carotene**	**µg**	2525.6	1686.5	2512.7	1694.8	0.576
**Carotene - total (carotene equivalents)**	**µg**	3016.7	1927.8	3009.1	1933.0	0.785
**Vitamin A - retinol**	**µg**	1542.1	2007.2	1505.7	2025.4	0.805
**Vitamin A - retinol equivalents**	**µg**	2048.2	2060.4	2010.4	2089.9	0.798
**Vitamin B2 - riboflavin**	**mg**	2.4	0.9	2.4	0.9	0.987
**Vitamin B1 - thiamin**	**mg**	1.8	0.8	1.8	0.8	0.200
**Vitamin B12 - cobalamin**	**µg**	9.6	6.4	9.5	6.4	0.794
**Vitamin B6 - pyridoxine**	**mg**	2.4	1.1	2.4	1.1	0.833
**Total folate**	**µg**	303.2	159.7	304.7	158.3	0.670
**Niacin**	**mg**	28.0	13.1	28.1	12.6	0.731
**Vitamin C - ascorbic acid**	**mg**	137.9	124.2	137.7	123.4	0.949
**Vitamin D - ergocalciferol**	**µg**	3.4	1.9	3.3	1.8	0.526
**Vitamin E - alpha tocopherol equivalents**	**mg**	14.7	8.4	14.9	8.3	0.406
**Zinc**	**mg**	11.4	4.8	11.3	4.7	0.838
**Nitrogen**	**g**	17.0	7.6	17.1	7.3	0.749
**Selenium**	**µg**	79.9	34.1	80.9	32.9	0.434
**Phosphorus**	**mg**	1702.1	659.5	1713.4	648.9	0.501
**Magnesium**	**mg**	322.3	149.7	324.4	146.7	0.585
**Manganese**	**mg**	2.9	1.8	2.9	1.8	0.909
**Sodium**	**mg**	3698	1616.5	3711	1601	0.371
**Iodine**	**µg**	184.3	72.0	187.3	71.5	0.061
**Potassium**	**mg**	3893.9	1810.8	3893.7	1805.3	0.476
**Calcium**	**mg**	1130.9	395.2	1134.4	392.2	0.051
**Chloride**	**mg**	5451.8	2414.8	5554.2	2374.8	0.319
**Copper**	**mg**	1.8	1.2	1.7	1.2	0.742
**Iron**	**mg**	11.9	5.3	11.8	5.2	0.620

*paired *t* test on log-transformed data.

## Discussion

The process of translation and adaptation of the EPIC-Norfolk FFQ yielded a Croatian version of the FFQ, which was very similar to the original questionnaire. The composition of items on the food list was carefully analyzed, with the aim of calculating the usual nutritional intake. The EPIC-Norfolk FFQ has been previously validated and widely used (13-15), therefore, we did not validate it again. The reliability analysis showed that the translated FFQ was a reliable method to assess diet in a self-administered way among children, adolescents, young adults, and pregnant women, with good reproducibility in young adults for almost all nutritional intakes. The group of young adults (N = 61) consisted mostly of college students of both sexes. The second group consisted of pregnant women, who were regarded as a separate group because of specific nutritional needs during pregnancy (21). The third group included children and adolescents. In childhood and adolescence, adequate nutrition is crucial for achieving full growth potential (22). Samuelson et al found that adolescents in Nordic countries had an irregular meal pattern; many of them skipped breakfast and school lunch but had dinner. However, snacking and light meals were very common, contributing to 25%-35% of the daily energy intake (23). This could lead to problems with weight control and consequent health issues.

The reliability of the Croatian FFQ was satisfactory for all groups, with Cronbach alpha values around 0.8-0.9 (18). The EPIC Norfolk FFQ was originally intended for the adult population in the UK and was previously validated for children and adolescents in Italy (10), Lebanon (11), Kazakhstan (24), and Romania (25).

The mean reproducibility was also satisfactory. Reproducibility is defined as a consistency of measurements on more than one administration to the same person at different times (26). ICC values for most of the nutrient intakes were between 0.75 and 0.9, which indicates good reproducibility (27). According to a meta-analysis by Cade et al (7), correlation coefficients between two administrations of a FFQ usually range from 0.5 to 0.7. The higher correlations found in the present study could be explained by our use of the Spearman correlation.

The interval between two administrations of FFQ in this study was three months. The interval between two administrations should be long enough for respondents to forget the answers given in the first administration and not too long so that dietary intakes do not change. Previous studies reported intervals ranging from one week to two years (28). Increasing the length of the interval between two administrations was found to decrease correlation coefficients (7). We believe the interval between administrations in this study was appropriate and long enough for respondents not to remember the answers from the first administration.

According to the meta-analysis by Cui et al (28), an FFQ with a correlation coefficient greater than 0.5 for most nutrients is considered a reliable tool to measure dietary intake. Accordingly, the Croatian version of the EPIC-Norfolk FFQ from this study could be regarded as a reliable questionnaire. A lack of significant differences in nutrient intakes assessed by FFQ1 and FFQ2 presented by Bland-Altman plots confirms this conclusion. The plots represented excellent agreement between FFQ1 and FFQ2, since most of the differences between measurements were very close to zero, and the mean differences were also near zero. Confidence intervals were affected by outliers, and were thus wider than desirable, but the position of the results indicated a small difference between the two administrations of the questionnaire. The reason for the presence of outliers is unknown.

Although in this study the FFQ was culturally adapted for Croatia, it could be used in the neighboring countries with similar language and dietary habits. Balkan region (countries of the former Yugoslavia: Slovenia, Croatia, Bosnia and Herzegovina, Montenegro and Macedonia, Romania, Bulgaria, part of Turkey, Greece, and Albania) (29-31) have similar dietary habits. The traditional food pattern in these countries is similar to the Mediterranean diet, with a distinct influence of western cuisine and food processing industry (29). Although the Croatian language is similar to the languages spoken in neighboring countries (Serbia, Bosnia and Herzegovina, Montenegro) (32), each language developed through time, and some terms in the Croatian language used in this FFQ could differ from terms in other similar languages in the region. However, most people are familiar with linguistic peculiarities of languages spoken in other countries in the region. This makes the possibility for misunderstanding of terms used in this questionnaire negligible. In order to facilitate the use of this questionnaire in other countries in the region it would be appropriate to the clarify food list and eventually include additional colloquial terms for some foods in the food list.

Our study has several strengths. We included the usual dishes from Croatian cuisine, but kept them as similar as possible to the original questionnaire to facilitate result calculation, and international comparisons. We followed the strict protocol to ensure the FFQ was correctly translated to Croatian.

This study also has some limitations. The questionnaire was administered for the second time only to the group of young adults. The main reason was that young adults have the most stable dietary habits among the respondents, as the other two groups were more likely to change eating habits in the test-retest period. Also, it was reported elsewhere that recruiting adolescents can be quite challenging (33), and the sample size of 50 is acceptable for a validation study (7). Finally, since the majority of the respondents were college students and well-educated women, self-administration of this questionnaire to a population with a lower educational level could lead to problems with understanding the questionnaire instructions. The use of this questionnaire in general population would probably require the administration by interviewers instead of self-administration.

In conclusion, this adaptation and reliability study showed that the Croatian FFQ adapted from the EPIC-Norfolk FFQ had good reliability calculated as internal consistency for all included subgroups, and good reproducibility for most nutrients for young adults.
